# Supplementary material for: Are carbon emissions trading and green financial instruments synergistic? -Comprehensive quantitative research based on content analysis
Source: PLoS One. 2024 Mar 7;19(3):e0298601. doi: 10.1371/journal.pone.0298601 (PMC10919601; doi:10.1371/journal.pone.0298601)
Supplement: S1 Data — (ZIP) [file pone.0298601.s001.zip › Data/地级市Z_F工作报告.zip!/║■▒▒/╧╠─■╩╨/└┤╫╘╣1⁄2╓┌║┼í╛┬φ┐╦╩2╛▌═°í┐_2011.docx]

各位代表：

　　现在，我代表市人民政府向大会报告工作，请连同《咸宁市经济和社会发展第十二个五年规划纲要（草案）》一并审议，并请各位市政协委员和列席人员提出意见。

　　一、2010年及“十一五”时期的工作回顾

　　2010年，在省委、省政府和市委的坚强领导下，全市上下深入贯彻落实科学发展观，远谋近施，真抓实干，推动经济社会又好又快发展。实现生产总值520.33亿元，增长15.9%；规模以上工业增加值206.04亿元，增长25.7%；全社会固定资产投资436.29亿元，增长44.7%；社会消费品零售总额205.04亿元，增长28.7%；出口总额1.68亿美元，增长78.4%；实际利用外资1.54亿美元，增长37.4%；全地域财政收入79.59亿元、地方财政总收入39.13亿元、地方一般预算收入23.34亿元，分别增长44.1%、21.9%和28.7%；城镇居民人均可支配收入12968元、农民人均纯收入5606元，分别增长11.5%、15%；城镇新增就业3.9万人，城镇登记失业率3.98%；人口自然增长率5.87‰。节能减排任务全面完成。

　　2010年各项目标任务的圆满完成，标志着“十一五”时期我市经济社会发展驶进了快车道，跨入了转型期。

　　过去的五年是极不平凡的五年。办成了一些大事，办妥了一些难事，办好了一些喜事。科学确立了鄂南经济强市建设的战略目标，圆满完成了深入学习实践科学发展观活动，有效应对了国际金融危机的冲击，努力战胜了雨雪冰冻、暴雨洪涝等重大自然灾害，成功举办了两届国际温泉文化旅游节，隆重纪念了改革开放30周年暨撤地建市10周年，超额完成了“十一五”规划确定的主要目标任务。

　　过去的五年是积极作为的五年。在解放思想、攻坚克难中奋力赶超，在抢抓机遇、砥砺前行中创先争优。生产总值是“十五”期末的2.5倍，前四年增速分别居全省第5位、第3位、第1位和第1位，年均增长15.6%。

　　三次产业比重由“十五”期末的26.4：39.6：34调整为19.4：46.5：34.1。全地域财政收入、地方财政总收入、地方一般预算收入分别是“十五”期末的4.2倍、3倍和3.1倍，年均分别增长33%、24.2%和25.7%。

　　过去的五年是夯实基础的五年。坚持应急与谋远结合、共识与共为并重，努力做好打基础、利长远的工作。三次召开建设鄂南经济强市座谈会，凝聚了智慧与力量，创新了思维与举措。五年完成固定资产投资1181亿元，是“十五”时期的3.6倍，基础设施、产业体系不断完善。823个项目列入省项目库。立体化、网格化的交通格局初步形成，新增通车里程8473.5公里，是“十五”时期的1.8倍；耕地占补平衡有余，新增耕地面积10万亩；110平方公里的城市土地利用总体规划获批，“五城二区”建设全面启动。城镇化进程加快，城镇化率由“十五”期末的42.4%提高到49.3%。

　　过去的五年是共建共享的五年。正确处理财力有限与民生事大的关系，加大投入助民、保障惠民力度。五年用于教育、科技、文卫、社保的财政支出分别是“十五”时期的3.5倍、3.2倍、6倍和7.5倍。五年新增城镇就业18.9万人，城镇登记失业率控制在4.1%以内。坚持每年办好十件实事，一批群众关心的民生问题逐步解决。城镇居民人均可支配收入、农民人均纯收入分别是“十五”期末的1.8倍、1.9倍。

　　五年来，我们经受了复杂形势的考验，收获了弯道超越的喜悦。

　　（一）坚持不懈地扩大有效需求。加大项目建设力度。五年争取中央投资40亿元，财政安排投资99亿元，带动民间投资603亿元，分别是“十五”时期的2.6倍、2.3倍和4.3倍；咸宁核电、林浆纸一体化等一批重大项目前期工作基本完成，赤壁电厂二期、咸黄高速、通界高速及市博物馆、青少年宫、传媒大厦等项目开工建设，武咸城际铁路、武咸快速通[FS:PAGE]道、杭瑞高速和大广高速咸宁段、咸通高速等项目加紧推进，红牛一二期、光宝电子一期建成投产，天然气管道全线贯通，武广高速铁路正式通车，赤壁市、嘉鱼县、咸安区挤进武汉新港建设规划。2010年全市8个省级开发区（工业园）完成投资150亿元，实现税金17亿元，分别是“十五”期末的9.6倍、3.9倍，其中咸宁经济开发区完成投资36.2亿元，实现税金4.1亿元，分别是2006年的5倍、12.5倍。积极推动消费转型升级。城市、农村商品零售额分别达172.31亿元、32.73亿元，是“十五”期末的3.4倍、1.3倍。居民消费价格基本稳定。金融机构存贷款余额分别达432.52亿元、226.88亿元，是“十五”期末的2.8倍、3倍。保险费收入12.8亿元，移动电话用户157.8万户，宽带用户19.1万户，邮政通信业务总量11.6亿元，分别是“十五”期末的2.9倍、3.9倍、4.3倍和1.9倍。

　　（二）坚持不懈地推动产业优化升级。巩固农业基础地位，大力发展特色农业、规模农业、品牌农业、效益农业，有效实施“山上再造”工程，农业结构不断优化。粮食生产连年丰收，总产达22.4亿斤，主要农产品增产增效。年销售收入过亿元和省级农业产业化龙头企业分别发展到16家、26家，优质农产品基地331万亩，“三品”标志认证275个。强化工业主导作用，着力壮大支柱产业、优势企业，加快培育新兴产业、中小企业。规模以上工业企业807家，比“十五”期末净增451家。工业利润和税金年均分别增长38.9%和26.1%。电力能源、纺织服装、食品饮料、机械电子、森工造纸、冶金建材等六大支柱产业工业总产值478亿元，是“十五”期末的5.4倍。高新技术产业增加值15.1亿元，年均增长23.6%。省级名牌产品、著名商标分别达36个、30个，联乐床垫荣获“中国名牌产品”称号，川字牌砖茶、澳森牌地板荣获“中国驰名商标”称号。加快发展以旅游休闲为主体的服务业，服务业增加值177.4亿元，年均增长17.1%。五年接待入境游客3202万人次，实现旅游总收入138.9亿元，咸宁跻身国家首批旅游标准化试点五市之列。房地产投资59.6亿元，是“十五”期末的15.7倍。花旗银行、交通银行、民生银行、吴江农村商业银行、黄石银行等金融机构和武商、中百、中商、银泰、恒信德龙等知名商家进驻咸宁，4家小额贷款公司成功组建，中介、休闲、信息、物流等新兴服务业竞相发展。

　　（三）坚持不懈地深化改革开放。不断深化以产权制度改革为重点的国有企业改制，稳步推进市直机关所办企业脱钩改制，国有资产监管得到加强。加大民营经济扶持力度，发展各类市场主体85024户，注册资金379亿元，分别是“十五”期末的1.6倍、3.1倍。稳步推进“两型”社会综改试验，三年行动计划基本完成，厅市合作成效明显。市、县两级政府机构改革基本完成。事业单位分类改革走在全省前列。深化农村综合配套改革，公益性服务“以钱养事”机制不断完善，“小三场”和集体林权制度改革有序推进。加快金融体制改革，金融机构11家，比“十五”期末增加7家，每年荣获“湖北省金融信用市”称号。规范土地市场，五年实现土地交易额90亿元。财税体制改革不断深化。医疗卫生体制改革稳步推进。教育、科技、文化和住房体制等方面改革取得重要进展。不断优化发展环境，下放、取消、调整行政审批事项465项，咸宁被评为全国“浙商（省外）最佳投资城市”。深化开放合作，五年出口总额5.1亿美元，吸引外商直接投资4.96亿美元，分别是“十五”期间的1.8倍、3倍；承接产业转移成果丰硕，五年引资项目2409个，到位资金412.5亿元。泰国红牛饮料、香港华润电力、台湾统一饮品、光宝电子、韩国大宇汽车、广东碧桂园地产、山东晨鸣纸业等一批国内外知名品牌落户咸宁。

　　（四）坚持不懈地统筹城乡发展。不断优化城市空间布局，城市总体规划修编全面完成。市区一[FS:PAGE]体化发展的运行机制不断完善，以“五城二区”建设为主体的市区发展框架初步拉开。五年投入建设资金149亿元，淦河综合治理、潜山国家森林公园、城区干道沥青化、人行道改造、南外环、青龙路、麦笠山路、桂乡大道等一批重点工程顺利竣工，社区建设、立面整治、拆违治超等工作成效显著，“五城同创”深入开展，城市经营管理机制不断创新，城市功能和容量不断提升。以临江工业园和临江重点镇建设为重点的沿江经济带、以特色经济板块为重点的沿路经济带建设全面启动。县域经济活力增强，咸安机电产业、苎麻纺织产业集群、赤壁纺织服装产业集群、通城涂附磨具产业集群、通山石材产业集群等五大产业集群被确定为省重点成长型产业集群，县域规模以上工业增加值180亿元、地方一般预算收入16.9亿元，分别是“十五”期末的4.6倍、2.8倍。重点镇和新农村建设稳步推进，“百镇千村”示范工程建设成效明显。通村油路覆盖率由“十五”期末的9%提高到100%。五年新建户用沼气池7.98万口，完成改厨改厕改圈6.83万户，解决了91.54万人的饮水安全问题。加快提升环境质量，建成一批污水处理厂和垃圾无害化处理场，污水处理率70%，垃圾无害化处理率30%；市区建成区绿化覆盖率37.7%。加大节能减排力度，五年依法关闭“五小”企业100家，万元生产总值能耗降低20%以上，化学需氧量和二氧化硫排放量分别下降3%、17.5%。中心城区环境空气质量优良率97.8%。咸宁荣膺省级园林城市、卫生城市、文明城市称号和中国人居环境范例奖，跻身全国最适宜人居的200个城市之列。

　　（五）坚持不懈地发展民生事业。五年帮助城镇失业人员就业再就业2.95万人，登记转移农村劳动力27万人。五险参保总数152万人次，是“十五”期末的2.4倍，企业退休人员基本养老金实现翻一番；常年生活救助对象50万人，城乡低保规模稳定在21万人左右；农村“五保”对象集中供养率68%；五年大病医疗救助人数11540人，临时救助城乡困难群众15454人次。五年新增廉租住房保障22180户、经济适用房保障2100户、农村危房改造7442户。城乡居民人均居住面积36平方米和39平方米，比“十五”期末分别提高5平方米和7平方米。解决了13.1万人的温饱和脱贫问题，完成了418个重点贫困村整村推进任务，扶贫搬迁1388户。城乡义务教育学杂费全面免收，义务教育学校绩效工资全面兑现，贫困学生资助体系逐步完善，各类教育稳步发展。五年取得省级以上重大科技成果38项，申请专利1700件，实施重大科技计划项目21项，6个县（市、区）均被列入全省科技富民强县计划。建成县级文化信息共享工程6个、乡镇综合文化站64个、村级农家书屋632个，《梦寻咸宁》、《白云深处》、《千古风流》、《隽水天城》、《千桥流水桂花香》等地域文化精品纷呈涌现。投入10.98亿元建设市中心医院、中医医院和6个县（市、区）医院、72个分片（农场）卫生院、430个村卫生室，为城乡居民免费提供9项基本公共卫生服务；新型农村合作医疗实现全覆盖。手足口病、艾滋病、血吸虫病等重大传染病防治取得实效。人口计生工作获省表彰。社会科学、文学艺术、新闻出版、广播影视事业加快发展。抗震救灾对口支援任务提前完成，援疆工作全面启动。驻咸解放军和武警部队在抗灾救灾、维护稳定、支持发展中作出了突出贡献，民兵预备役工作取得新进步。民族、宗教、对台、外事、侨务等工作扎实推进。人防、保密、气象、水文等工作得到加强。统计、物价、档案、方志等工作迈出新步伐。妇女、儿童、老龄、慈善、残疾人等事业全面发展。人民团体和社会组织的作用有效发挥。群众性精神文明创建活动深入开展，公民思想道德素质和城乡文明程度不断提高。

　　（六）坚持不懈地加强政府自身建设。自觉坚持党的领导，自觉依照法定权限和程序行使权力、履行职责，[FS:PAGE]自觉接受市人大法律监督、工作监督和市政协民主监督，自觉听取各民主党派、工商联和无党派人士意见，五年办理人大议案建议361件、政协提案618件，办复率100%，满意率95%以上。扎实推进“能力建设年”、“作风建设年”活动，不断完善绩效考核，政府执行力不断提升。优化行政审批，加强电子政务建设，推进政府信息公开，行政效能不断提高。积极开展文明执法教育活动，行政执法行为不断规范。不断完善政府决策机制，建立健全专家咨询制度和合法性审查制度。强化行政监察和审计监督，加大部门和行业不正之风整治力度，扎实推进重点领域专项治理，严肃财经纪律，政府系统党风廉政建设取得较好成效。基层民主实现形式不断丰富。“五五”普法依法治理工作全面完成。应急救援和减灾防灾体系逐步健全，信访、维稳工作机制进一步完善，食品药品安全得到有效保障，安全生产、社会治安形势总体趋好。

　　回顾过去的五年，我们深切地体会到，咸宁作为后发地区，要实现赶超发展、跨越发展，必须坚持以科学发展观为指导，好中求快，能快则快；坚持以解放思想为先导，深化改革，全面开放；坚持以项目建设为支撑，扩大内需，调整结构；坚持以改善环境为保障，改进作风，提升效能；坚持以服务群众为宗旨，改善民生，促进和谐。

　　回顾过去的五年，我们清醒地认识到，全市经济总量还不大，结构还不优；工业集群化、农业产业化、服务业现代化和市域城镇化程度还不高；全民创业氛围还不浓，城乡居民增收渠道还不宽；城市配套功能还不强，中心城区优质教育资源配置不尽合理和停车难、公厕少等问题亟待解决；政府转型建设还不快，公务员能力建设和作风建设有待进一步加强。这些问题需要我们以高度负责的态度，采取切实有效的措施，逐步加以解决。

　　各位代表，在历届党委、政府的领导下，咸宁正在发生深刻而生动的变化，发展理念不断创新，体制机制不断完善，经济实力不断增强，生机活力不断显现！所有这些，应归功于上级党委、政府和市委的坚强领导，归功于市人大、市政协的监督支持，归功于全市上下的团结拼搏，归功于社会各界的热情参与。在此，我谨代表市人民政府，向全市广大干部群众，向各位人大代表、政协委员和各民主党派、工商联、无党派人士，向离退休老同志，向驻咸解放军、武警官兵和政法干警，向中央、省驻咸单位，向所有关心支持咸宁发展的社会各界人士和海内外朋友，表示最崇高的敬意和最衷心的感谢！

　　二、“十二五”时期的总体部署

　　“十二五”时期是咸宁推动科学发展、跨越发展、和谐发展的重要机遇期，是全面建设小康社会的关键期，是实现鄂南经济强市建设目标的攻坚期。市委三届十次全会通过的《关于制定咸宁市经济和社会发展第十二个五年规划的建议》，提出了未来五年的奋斗目标、指导方针和主要任务。根据《建议》精神，市政府制定了《咸宁市经济和社会发展第十二个五年规划纲要（草案）》。《纲要》审议通过后，我们将认真组织实施。

　　“十二五”时期，咸宁的发展既面临新的重大考验，又面临难得的历史机遇。总体上机遇大于挑战。机遇来自我国工业化、信息化、城镇化、市场化、国际化的深入发展，来自宏观调控的倒逼机制，来自中部崛起、“两圈一带”等战略的大力实施，来自市内一批重大产业、基础设施和民生项目的有力支撑，来自区位优势、资源优势、环境优势的叠加效应。我们不仅有区域发展的大环境，还有后发赶超的小气候，既有率先步入高铁时代、率先开通城际铁路等交通优势，又有内陆首家核电、华中最大火电厂等产业优势，还有资源环境承载力等基础优势，完全可以融入大武汉，前接珠三角，东联长三角。机遇珍贵，稍纵即逝。我们务必珍惜机遇、抢抓机遇、用好机遇，顺势而为、乘势而上，推动经济[FS:PAGE]社会更好更快发展。

　　“十二五”时期全市经济社会发展的总体要求是：以邓小平理论和“三个代表”重要思想为指导，深入贯彻落实科学发展观，紧紧围绕建设鄂南经济强市总目标，以科学发展为主题，以加快转变经济发展方式为主线，以提高城乡人民生活水平为根本出发点和落脚点，以推进新型工业化、新型城镇化和现代服务业、现代农业为重点，以改革开放创新为动力，进一步解放思想、抢抓机遇，大力实施“工业崛起、旅游跨越、城乡统筹、生态持续、人才保障”五大战略，着力打造小康咸宁、生态咸宁、宜居咸宁、健康咸宁、和谐咸宁，推动经济社会跨越发展，为全面建成小康社会打下坚实基础。

　　各位代表，我们把解放思想、跨越发展写在咸宁“十二五”发展的旗帜上。解放思想是关乎咸宁跨越发展的总开关，是干事谋事的逻辑起点，是科学发展的第一动力。发展不够是咸宁最大的实际。只有继续解放思想，进一步深化体制机制的改革创新，建立发展的统一战线，营造有利于发展的人文环境，才能开拓更宽的发展视野、更大的发展空间、更多的发展资源，切实解决咸宁发展中的重大问题，抢占科学发展的先机，实现跨越发展的目标。《纲要（草案）》提出“两高一好、两个明显”的总体要求：全市经济增长速度高于全省平均水平、高于武汉城市圈平均水平，发展质效好于全市“十一五”时期，综合实力明显提升，在全省位次明显前移；“六个翻一番、四个不低于”的预期目标：生产总值、规模以上工业增加值、地方一般预算收入、社会消费品零售总额、城乡居民收入、中心城区建成区面积在“十一五”的基础上翻一番，即生产总值突破1000亿元、规模以上工业增加值突破400亿元、社会消费品零售总额突破500亿元、地方财政总收入100亿元、城镇居民人均可支配收入24000元、农民人均纯收入10000元、中心城区面积100平方公里左右，全市城镇化率、社会保障水平不低于全省平均水平，万元生产总值能耗、化学需氧量和二氧化硫等主要污染物排放量下降不低于省节能减排要求的下降比例；“七地一区”的创争目标：努力把咸宁建成全国新型能源基地、全国优秀旅游目的地、中部地区产业转移重要承接基地、全省区域性现代制造业和高科技研发转化基地、全省区域性现代商贸物流基地、全省现代农业示范基地、武汉城市圈“两型”社会建设综合配套改革创新示范基地和全省首家低碳经济示范区。这个总体目标，既考量了咸宁目前的发展基础与增长水平，又体现了“努力于快、服从于好、又好又快”的发展要求；既顺应了省委、省政府要求咸宁加快建设鄂南经济强市的殷切期望，又顺应了咸宁人民过上更好生活的共同期盼。“十二五”时期，我们将以后发抢先的胆识、积极担当的精神，力争早日建成鄂南经济强市，不断开创咸宁科学发展的新局面。

　　（一）坚定不移地推动内生增长。保持投资增长强度，优化政府投资，拓展民间投资，促进直接融资，依靠重大项目带动投资较快增长，力争累计投资4400亿元。拓展消费需求，积极实施扩大消费政策，促进消费升级，培育消费热点，提升消费能力，改善消费预期，力争全社会消费品零售总额年均增长20%。加速工业崛起步伐，大力推进工业园区建设，推动咸宁经济开发区建成国家级开发区；坚持合作共建，大力建设沿江、垂江经济带；着力打造电力能源、冶金建材、森工造纸、纺织服装、机电制造、食品饮料等6个年产值过百亿元的支柱产业，加快发展高新技术产业和战略性新兴产业。大力发展现代农业，突出发展高产、优质、高效、生态、安全农业，实现农业产业化、规模化、标准化和集约化，推动新农村建设纵深发展。加快现代服务业发展，改造提升餐饮娱乐、交通运输等传统服务业，加快发展电子商务、连锁经营等现代营销业，做大做强旅游、信息、文化、物流、金[FS:PAGE]融等现代服务业，有序发展房地产业。到“十二五”期末，力争旅游总收入占GDP比重达16%以上，三次产业比重达12：48：40。

　　（二）坚定不移地深化改革创新。全面推进重点领域和关键环节的改革，加快构建充满活力、富有效率、更加开放、有利于科学发展的体制机制。把握先行先试主动权，突破性地推进“两型”社会建设综合配套改革。创新土地管理、投融资、户籍管理和社会保障机制。深化行政管理体制改革。稳妥推进教育、科技、文化、卫生等社会事业分类改革。推进科技创新，健全以市场为导向、以企业为主体、产学研结合的创新体系。面向经济社会发展的科技需求，突破一批关键技术，开发一批重点产品，推广一批先进技术，新建一批农业科技创新示范基地，促进经济发展方式转变，科技事业在全省位次明显前移，力争省级以上研发机构达20家，取得各类科技成果200项，开发新产品200项，专利申请量达2000件。坚持“服务发展、人才优先、以用为本、创新机制、高端引领、整体开发”的方针，统筹各类人才队伍建设，形成引得进、留得住、用得好的人才工作机制。

　　（三）坚定不移地扩大开放合作。全面融入开放大潮，真开放，先开放，大开放，全开放，形成人人都是开放精神的践行者、开放成果的创造者的生动局面。全面提升开放实效，坚持引资与引智相结合，加强与武汉城市圈、长株潭城市群两大“两型”社会建设综改试验区的深度融合，推进与珠三角、长三角的全面对接，积极承接国际国内产业转移；充分借用武汉、广州、上海等大城市要素配置优势，吸引更多的资金、技术、人才落户咸宁；大力发展总部经济，重视引进高端产业；鼓励支持5-7家企业上市；发挥区位交通优势，打造未来湖北物流中心。着力推进出口增长，加快出口产业带建设，优化出口商品结构，支持优势企业境外投资。

　　 （四）坚定不移地统筹城乡发展。牢固树立全域发展理念，以“两纵三横”为纽带，以推进新型城镇化为突破口，加快推进城乡一体化建设，形成城乡规划、产业布局、基础设施、公共服务、社会管理一体化的发展格局。做大做强中心城市，抓好城乡一体化建设试点，率先建成改革开放先行区、城乡统筹和“两型”社会建设示范区。加快发展县域经济，促进工业园区化、产业集群化、城镇特色化发展。大力培育发展中心镇，建成一批工业型、农业型、商贸型、旅游型、资源开发型的特色镇，发展成为县域经济次中心。加快基层基础建设，推动中心村建成城镇化居民点，促进社区建设功能化、管理网格化。

　　（五）坚定不移地推进低碳发展。树立低碳、绿色、循环发展理念，建设“两型”社会，增强可持续发展能力。加快发展低碳经济，打造全省首个低碳经济示范区，建设一批低碳工业园和低碳产业项目。发展绿色建筑，创建全国可再生能源建筑示范城市。抓好高耗能行业和企业节能，促进农村低碳化建设，提高水、土地、矿产等各项资源的使用效率。加快发展循环经济，支持循环经济技术研发和推广。实施绿色生态工程，创建国家园林城市和环境保护模范城市。开展重点流域水生态保护与修复工程建设，加强饮用水源地保护。加强对污染物排放的控制。倡导绿色消费，促进绿色增长，实现经济效益与生态效益的有机统一。

　　（六）坚定不移地发展民生事业。坚持民生优先，完善公共服务体系，推进基本公共服务均等化，让人民生活得更加幸福、更有尊严。实施积极的就业政策，规范劳动就业市场，落实就业扶持政策，完善就业援助制度，鼓励自主创业。逐步建立全覆盖、可选择、多层次、能转接的城乡一体社保管理和服务体系。加快公租房、廉租房、经济适用房建设和棚户区、农村危房改造，提高住房保障水平。完善城乡居民最低生活保障制度，实现应保尽保。完善各项专门救助制度。推进公共财政建[FS:PAGE]设，加大民生事业投入。深化文化体制改革，推动文化事业与文化产业互动发展，提升文化软实力。加强公民思想道德建设。创新社会管理模式，完善信访维稳和安全保障机制，巩固和发展安定和谐的社会局面。

　　三、2011年的工作安排

　　今年是“十二五”的开局之年，是实现新一轮跨越发展的基础之年，是加快转变发展方式的关键之年，是中国共产党成立90周年。做好今年的政府工作，意义深远，责任重大。

　　全市经济社会发展的主要预期目标是：生产总值增长15%以上，规模以上工业增加值增长22%，全社会固定资产投资增长30%，社会消费品零售总额增长20%，吸收外商直接投资增长10%，外贸出口总额增长15%，地方一般预算收入增长20%，城镇居民人均可支配收入增长13%，农民人均纯收入增长13%，居民消费价格涨幅控制在4%左右，城镇登记失业率控制在4%以内，人口自然增长率控制在6‰以内，万元生产总值能耗下降3%，化学需氧量和二氧化硫排放量分别下降1.5%、1%。

　　（一）推动经济运行在加快转型中提速提质

　　着力扩大内需。努力增加投资，健全重点项目推进机制，重点抓好100个投资过亿元的项目建设，力争全社会固定资产投资570亿元以上。加快推进以咸宁核电、赤壁电厂二期、咸通高速、通界高速、咸黄高速、嘉鱼长江大桥、杭瑞高速咸宁段、潘家湾码头等八大项目为重点的基础设施建设工程，推动林浆纸一体化、赤壁长江大桥、皇明太阳谷、南玻节能玻璃等重大项目取得重要进展。注重优化投资结构，引导投资投向现代农业、先进制造业、现代服务业、战略性新兴产业、生态环境保护和社会事业等重点领域。抓住国家加快水利改革发展的重大政策机遇，大力发展民生水利，实施好农村饮水安全、病险水库加固、泵站改造、灌区配套等建设工程，确保水利投资大幅增长。加强项目库建设，争取更多的重大项目纳入国家、省投资目录。全面促进城乡消费，进一步落实好鼓励消费的政策措施，有效提升消费能力，努力开拓消费市场，不断优化消费结构，力争社会消费品零售总额达250亿元。加快推进以咸宁游客接待中心、大型商业综合体、温泉乐园、金桂湖国际休闲度假中心、嘉鱼三湖连江风景区、赤壁龙佑温泉二期、九宫山旅游度假中心、崇阳浪口温泉等八大项目为重点的消费设施建设工程，大力加强专业市场、高端商场和特色商业街、星级酒店、社区便民街、乡村农家店建设。继续实施社区“双进”工程，扩大“万村千乡”、“双百”、“新网”等工程覆盖面，新建村级综合服务社180个。大力推进“三下乡、两换新”力度，服务农村消费，繁荣农村市场。积极发展电子商务和连锁经营。强化市场监管，让群众放心消费、便利消费。

　　不断提高产业竞争力。加快工业集群发展步伐。做强支柱板块，推动六大支柱产业加快发展，确保六大支柱产业总产值占全市工业的比重达80%。做大创新板块，着力培育发展新能源、新型环保材料、电子信息、船舶制造、生物医药等新兴产业和高新技术产业，深入开展科技投融资、产学研合作、创新型企业建设、农业科技创新、中小企业成长路线图计划等专项行动，加强创业服务平台、科技创新平台建设，力争新兴产业的主要指标增幅不低于规模以上工业增加值增幅、高新技术产业增加值增长20%。做优活跃板块，支持各级开发区加快发展，促进工业园区化、园区产业化、产业集群化，确保园区工业总产值增幅均高于25%，咸宁经济开发区主要指标增幅达35%。加大精品名牌培育力度，新增10个省级以上名牌产品、驰名商标。加强经济运行调度，抓好要素供应保障。加快发展现代农业，着力转变农业发展方式，大力发展规模种植、特色养殖、林下经济、观光农业，促进特色农业更特、优势农产品更优。继续抓好优质粮产业工程，力争农业总产值增长5%。支持咸安畜禽[FS:PAGE]、嘉鱼蔬菜、赤壁水产、通山水果、通城油茶、崇阳鸭业等特色基地建设，力争优势农产品基地发展到350万亩。深入推进“山上再造”、秋冬农业开发。主攻农产品精深加工，延长产业链、提高附加值，新增销售收入过10亿元的龙头企业5家。加快发展农民专业合作组织，不断提高农民组织化程度。积极推行“农超对接”等经营模式，切实搞活农产品流通。推进农业标准化生产，新增“三品”标志认证30个。突破性发展现代服务业。瞄准“双康”时代消费热点，大力发展旅游休闲业，打造城市旅游综合体，建设旅游主题街区。坚持以生态为基础、以文化为核心、以产业为引擎，加快整合旅游资源，不断充实旅游要素，健全旅游配套设施，开发旅游特色产品，推进旅游标准化建设，办好第三届国际温泉文化旅游节，力争接待游客达1400万人次，旅游总收入增长30%以上。积极发展楼宇经济、现代物流、金融保险、文化创意等服务业态，大力发展评估、咨询、物业等新兴服务业。促进房地产业稳健发展。推进电信网、互联网和广电网“三网融合”，发展数字物流，建设信息化示范市。

　　努力推进城乡协调发展。坚持以城带乡、城乡互动，以规划为龙头，以建管为重点，以产业为支撑，加快谋划和推进城乡一体化建设，力争城镇化率提高1.5个百分点。高昂市区发展龙头，进一步完善市区一体化发展体制机制，整合市区资源，加大“五城二区”建设力度，抓好咸安区城乡一体化建设试点，加快市区经济社会发展步伐，力争主要经济指标增幅高于全市平均水平5个百分点。强化规划龙头作用，城区控制性规划覆盖率达80%。拉大主城区框架，逐步完善综合交通、园林绿化、城市防洪、停车场所、公共厕所等配套设施，提高基础设施承载力、产业聚集配套力和市民生活保障力。创新城市管理机制，深入开展“五城同创”，大力整治违章建筑，努力缓解交通拥堵现状。加大城市经营力度，加快促进“城中村”、“城郊村”改造，提高土地、资产等资源的营运效益。推动县域经济协调更快发展。抓住长江经济带新一轮开放开发机遇，启动咸嘉临港新城建设，大力推进“北三县”中小城市群和“南三县”特色经济带建设，继续支持咸安抓好物流、纺织、森工等产业建设，支持嘉鱼抓好化工、建材、船舶制造等产业建设，支持赤壁抓好纺织服装、能源、造纸等产业建设，支持通城抓好涂附磨具、陶瓷、电子等产业建设，支持崇阳抓好汽车配件、钒业、医药卫材等产业建设，支持通山抓好乡村旅游、石材产业建设，推动县域经济特色化、规模化发展，综合实力在全省争先进位。积极推进新型城镇化，围绕夯实基础、培育产业、完善功能、提升品位，加快中心镇建设，力争1—2家进入全省30个镇级市试点。加强中心村建设，优化空间布局，集约配置资源，以点带面推进新农村建设。围绕推进城乡一体化建设，稳步推进户籍和社保制度改革。

　　大力加强生态建设。实行严格的节能减排工作责任制和问责制，强化能源资源节约和高效利用，开展先进节能技术开发、示范和推广，推进工业、交通、建筑三大重点领域节能。全面推行排污许可证制度，严格执行环境影响评价制度和建设项目“三同时”制度。加快危险废物集中处理中心、垃圾无害化处理工程建设，确保县（市、区）和咸宁经济开发区污水处理厂全面建成并投入运营。积极发展循环经济和低碳经济，加快发展清洁能源、环保等低碳产业，加大与省联发投、华彬国际集团合作力度，加快低碳经济示范区建设。鼓励社会各界碳汇造林。加大“两湖”、“三河”、“十库”等重点水域流域综合治理力度，开展生态公益林保护、退耕还林、自然山体水体保护、水土保持、用水水源地保护工作。建立健全防御极端气候变化的预警预报体系，增强处置气象灾害的能力。

　　（二）推动民生事业在共建共享中全面进步[FS:PAGE]

　　改善人民生活。认真落实收入分配制度，建立健全职工收入稳定增长机制，兑现津补贴政策，提高最低工资标准和最低生活保障线水平。千方百计扩大就业，鼓励和支持劳动者自主创业、自谋职业、灵活就业，重点解决好高校毕业生、零就业家庭、残疾人、低保对象、就业困难人员和返乡农民工就业再就业问题。加快完善社会保障体系，推进社保扩面工作，完善统筹城乡社会养老保险、城镇居民基本医疗保险和新型农村合作医疗体系，搞好新型农村养老保险试点，落实企业退休人员基本养老金调标、医疗保险提高待遇等工作，力争五险参保面达150万人次以上。坚持脱贫奔小康、城乡结对共建、开发式扶贫相结合，加快推进通山脱贫奔小康试点，落实好贫困地区和水库移民后期扶持政策，解决1.7万贫困人口稳定脱贫问题。

　　促进文化繁荣。本着公益、基本、均衡、便利原则，建立健全城乡公共文化服务体系。加强文艺精品生产，不断繁荣舞台艺术。推进文化惠民工程建设，开展送戏、送书下乡活动，丰富农村文化生活。挖掘、整理、传承民俗文化，加强文化遗产保护。推出更多理论成果和文化精品。深化文化体制改革，推进文化事业与文化产业协调发展。加快发展新闻出版、广播影视、文娱演艺、网络文化等重点产业，鼓励和引导社会资本参与文化产业，形成一批骨干企业。深入实施全民健身计划，大力推进健身网点建设，做好亚洲沙滩排球巡回赛承办工作。推进精神文明建设，加强社会科学普及和普法教育，提高公民思想道德素质。加强互联网等新兴媒体建设、运用、管理，把握正确舆论导向，提升传播能力。强化对外宣传，提升城市形象。

　　加强人才培养开发。牢固树立人才是第一资源的观念，创新人才培养、引进、使用、管理机制，坚持党政人才、专业技术人才、经营管理人才、高技能人才和农村实用人才等五支人才队伍一起抓。建立健全分级分类培训教育体系。健全柔性引才机制，做好急需的高层次技术人才、管理人才和复合型人才引进工作。进一步深化选人用人制度改革，逐步建立科学有效的人才评价体系和激励机制，激发用人单位在人才队伍建设中的积极性、创造性。

　　加快社会事业发展。进一步落实教育优先发展战略，优化教育布局和资源配置，加大教育投入力度，促进义务教育均衡发展，统筹发展学前教育、高中教育、成人教育和特殊教育，积极探索职业教育发展新机制，充分发挥高等教育服务地方经济发展的功能和潜力。提升科技创新能力，加快科技成果转化，促进科技入园、入村、入户，力争新认定高新技术企业增加2家、转化科技成果80项以上。整合农业科技资源，组建农业科学院。健全医疗服务体系，扎实做好医改工作，巩固完善新型农村合作医疗制度，稳妥推进国家基本药物制度建设，加快基层医疗卫生服务体系建设。

　　抓好艾滋病、结核病、血吸虫病等重大传染病的防治工作。稳定低生育水平，控制出生人口性别比，提高出生人口素质。进一步保障妇女、儿童、老龄和残疾人合法权益，积极发展社会福利、社会救助、优抚和慈善事业。扎实抓好档案、保密等工作。认真做好市、县两级地方志书编纂出版。进一步做好统计工作。加快推进以市博物馆、妇儿中心、青少年宫、传媒大厦、奥林匹克体育中心、十六潭公园、文化艺术中心、老年公寓等八大项目为重点的社会事业建设工程，不断提高公共服务水平。

　　创新社会管理。健全新型社区管理服务体制，提高社区自治能力和服务功能。扩大农村社区建设试点范围，推进农村社区化发展。依法做好第八届村委会换届选举工作。发挥人民团体在社会管理服务中的作用。建立健全社会应急管理机制，不断提高危机管理与风险防范能力，有效应对各类突发公共事件。强化安全生产监管，加强食品、药品、餐饮卫生监督，推进防震减灾体系[FS:PAGE]建设，确保人民群众生命财产安全。加强价格监测、预警和监管，抓好“米袋子”、“菜篮子”工程建设，保持居民消费价格基本稳定。大力整顿市场经济秩序，依法打击制假售假、商业欺诈等行为。健全信访联席会议制度，以群众工作统揽信访工作，加强社会治安综合治理，完善矛盾纠纷调解机制，深化“平安咸宁”创建活动。加强国防教育和双拥共建，支持驻咸解放军和武警部队建设，抓好民兵预备役和人民防空工作。进一步做好民族、宗教、对台、外事、侨务等工作。

　　切实为民办实事。在普惠、量力、当年的原则下，努力为城乡居民办好十大惠民实事建设工程。（1）城镇新增就业3.3万人，帮助农村富余劳动力3万人转移就业。（2）新建和筹集2826套廉租房和公租房，逐步解决教师住房困难。（3）确保新型农村社会养老保险适龄应参保人员达到32万人。（4）安排土地出让净收益的10%用于水利基础设施和城市防洪工程建设。（5）投入资金2亿元，推进农村环境保护连片整治试点。（6）筹措资金1亿元，支持城乡一体化建设。（7）市级财政安排300万元，支持县（市、区）村级卫生室建设，提高村级医生补助水平。（8）开工建设63公里咸安至崇阳生态旅游公路。（9）启动王英水库自来水工程建设，完成一号桥自来水厂泵站迁移工程。（10）依据市区教育布局规划，在市新城区改建一所九年一贯制学校。

　　（三）推动改革开放在解放思想中提升境界

　　深化重点领域改革。以先行先试的胆识和魄力深入推进“两型”社会建设综改试验，进一步创新体制机制，加快承接武汉产业转移，努力拓展厅市合作共建成果。深化国有企业股份制改造，推动骨干企业向优势行业和优势产业集中。稳妥推进党政机关与所办企业脱钩改制工作，完善国有资产经营预算制度，加强国有资产监管。进一步深化农村综合配套改革，不断完善公益性服务“以钱养事”机制，巩固发展集体林权制度主体改革成果。推进资源要素价格改革，促进资源产权和产品自由流动。加快推进金融产品和服务方式创新，吸引和支持金融机构进驻咸宁。积极培育上市后备资源，鼓励优势企业上市融资。完善县、乡财政转移支付制度，增强基层政府提供基本公共服务的能力。调整优化财政支出结构，优先保障“三农”、社会事业和民生工程投入。研究制定财税优惠政策，支持产业结构升级。优化税源结构，加强税收征管。执行最严格的耕地保护和节约集约用地制度，优化土地利用结构和布局，加强土地市场建设。深化医药卫生体制改革，建立四位一体、覆盖城乡的基本医疗卫生制度。

　　加大招商引资力度。抢抓武广高铁和武咸城际铁路开通机遇，优化发展环境，诚招天下客商，承接产业转移。加快对接沿铁路、沿江城市群和经济带建设，促进产业互动、资源互利、市场共享、合作共赢。按照招大、引强、选优的思路，瞄准“国”字号、行业100强、世界和全国500强企业，做好招商引资和择商引智工作，推动一批大项目、好项目落户咸宁。强化招商项目跟踪服务，提高项目履约率、见效率，力争引资到位资金增长20%，实际利用外资超过1.7亿美元。

　　大力促进全民创业。牢固树立“产业第一、企业家老大”的经济发展意识，把对外开放与对内搞活结合起来，建立健全政府鼓励、社会支持、市场调节的创业机制，完善促进全民创业的政策措施，引导高校毕业生自主创业，推动城乡居民灵活就业，支持各类企业再创业，让全民创业、自主创业成为人民群众的共同追求。毫不动摇地鼓励、支持、引导非公有制经济健康发展，进一步放宽市场准入，支持民间资本“非禁即入”，营造全民创业、“草根经济”蓬勃发展的土壤和气候，让一切劳动、知识、技术、管理和资本的活力竞相迸发。大力实施“中小企业成长工程”和“市场主体增量行动”，新增规模以上工业企业80[FS:PAGE]家，新增个体私营企业6000户。

　　积极发展对外贸易。适应国际市场和需求结构变化，着力培育一批出口龙头企业和企业集群，突出抓好以玉立砂带、三环方向机、友进钒业、大昇印染、华舟重工、汇昌皮革、石通石材、汇美达等八大企业为重点的出口基地建设工程，打造先进制造业出口基地，建设服务外包基地。巩固传统出口市场，开拓新型出口市场，优化出口商品结构，提高出口产品附加值和竞争力，力争外贸出口总额达2亿美元。鼓励有条件的企业境外投资。加快建立政府主导的外派劳务非经营性的综合基地，扩大对外劳务输出。努力改善通关条件，争取武汉海关和湖北出入境检验检疫局设立咸宁办事机构。

　　（四）推动政府服务在转变职能中优化改进

　　进一步转变政府职能。在创新社会管理和提供公共服务的同时，加强规划指导和经济运行分析调度，提高经济运行的稳健性；加强公共服务平台建设，健全服务网络，拓展服务领域，增强服务功能，形成广覆盖、多层次、社会化的服务体系；着力改善投资环境，激发创业创新活力，促进发展要素集聚；整顿规范市场经济秩序，完善行政执法、行业自律、舆论监督、群众参与相结合的市场监管体系；推进社会信用体系建设，建立健全信用监督管理和失信惩戒制度，强化社会诚信意识和诚信责任。尊重基层群众的首创精神，宽容探索中的失误，允许创新中的曲折，善待竞争中的失利，努力增创“精神区位”优势。

　　进一步规范行政行为。坚决贯彻落实市委各项决策，依法接受市人大法律监督、工作监督和市政协民主监督，认真办理人大代表议案、建议和政协委员提案，广泛听取各民主党派、工商联和无党派人士的意见建议，自觉接受新闻舆论和社会公众监督，坚持重大事项专家咨询、公众听证制度和规范性文件审查备案制度，做到科学决策、民主决策、依法决策。健全完善行政执法责任制、过错追究制、评议考核制，规范行政执法行为，完善行政执法程序。认真制定和实施“六五”普法依法治理工作规划。进一步清理行政审批事项，规范行政审批行为，完善行政审批机制。探索建立政务公开长效机制，拓展公开内容，创新公开形式，发展电子政务。

　　进一步改进政风行风。注重调查研究，深入基层解决实际问题，推动工作落实。注重提高效率，建立健全岗位目标责任、服务承诺、责任追究等制度，推行效能告诫、诫免教育等措施，真正实现以制度管事管人。注重投诉办理，加快效能投诉网络建设，建立效能投诉督查反馈、跟踪回访制度。注重精简会议文件，开解决问题的短会，发合法管用的文件。控制和规范检查、评比、达标活动。注重完善政风行风评议机制，深化创先争优活动，深入推进政务督查、效能监察、绩效考评，以效定绩，以为立位。

　　进一步推进廉政建设。进一步加强思想道德教育，引导政府公务人员讲政治、重品行、做表率。全面抓好“一岗双责”的落实，着力推进党员干部廉洁自律。深化“十个全覆盖”工作，加大源头治腐力度。深入开展专项治理，切实纠正损害群众利益的突出问题。健全政府采购、资产转让、项目招投标、土地招拍挂等管理制度，加强重点领域、关键岗位监管。继续抓好领导干部经济责任审计，加强专项资金使用、政府投资项目的审计和监察，严肃查处违法违纪案件。

　　各位代表！百舸争流，不进则退。唯有奋勇前行，方能不辱使命。让我们紧密团结在以胡锦涛同志为总书记的党中央周围，高举中国特色社会主义伟大旗帜，深入贯彻落实科学发展观，在省委、省政府和市委的坚强领导下，紧紧依靠全市人民，开拓创新，扎实工作，为早日建成鄂南经济强市，全面开创咸宁科学发展、跨越发展、和谐发展的新局面而不懈奋斗！
